# Comparison of preoperative two-dimensional shear wave elastography, indocyanine green clearance test and biomarkers for post hepatectomy liver failure prediction in patients with hepatocellular carcinoma

**DOI:** 10.1186/s12876-021-01727-3

**Published:** 2021-03-31

**Authors:** Rong Fu, Tingting Qiu, Wenwu Ling, Qiang Lu, Yan Luo

**Affiliations:** 1 Department of Ultrasound, West China Hospital of Sichuan University, Chengdu, 610041 Sichuan China; 2 Department of Ultrasound, West China Hospital of Sichuan University, No. 37, Guoxue Xiang, Chengdu, 610041 Sichuan China

**Keywords:** Ultrasound, Elastography, Hepatectomy, Liver failure, Indocyanine green

## Abstract

**Background:**

The preoperative prediction of post hepatectomy liver failure (PHLF) is essential, but there is no gold standard for the prediction at present, and the efficacy of different methods for the prediction has not been compared systematically. In this study, we aimed to compare the efficacy of preoperative two-dimensional shear wave elastography (2D-SWE), indocyanine green (ICG) clearance test and biomarkers for PHLF prediction in patients with hepatocellular carcinoma (HCC).

**Methods:**

We retrospectively studied 215 patients with HCC, who had undergone major liver resection in our hospital. Preoperative data of each patient, including liver stiffness value (LSV) of underlying hepatic parenchyma measured by 2D-SWE, ICG retention rate at 15 min (ICG-R15) measured by ICG clearance test, albumin-bilirubin (ALBI) scores, aspartate aminotransferase–platelet ratio index (APRI), and Fibrosis-4 (FIB-4) were collected for analysis. Post hepatectomy outcomes of study patients were also recorded for assessment of PHLF. The study patients were divided into development cohort (133 patients without PHLF, and 17 patients with PHLF) and validation cohort (59 patients without PHLF, and 6 patients with PHLF) randomly.

**Results:**

In the development cohort, LSV, ICG-R15 and ALBI scores were significantly different between patients with and without PHLF, while no significant difference of APRI and FIB-4 scores was found. LSV had higher AUC (the area under the receiver operating characteristic curve) (AUC = 0.795) for PHLF prediction than ICG-R15 (AUC = 0.619) and ALBI scores (AUC = 0.686) (*p* < 0.05 for all comparisons). In the validation cohort, the cutoff value of LSV obtained from the development cohort, 10.35 kPa,  revealed higher specificity (76.3%) for PHLF prediction than ICG-R15 (specificity: 66.1%) and ALBI scores (specificity: 69.5%) (*p* < 0.0001).

**Conclusions:**

Compared with ICG-R15, ALBI scores, APRI and FIB-4, LSV measured by 2D-SWE may demonstrate better efficacy for preoperative PHLF prediction in patients with HCC.

## Introduction

Liver resection is one of the main treatment options for hepatocellular carcinoma (HCC). However, post hepatectomy liver failure (PHLF) remains the major complication, which leads to the high risk of death [1]. Patients with HCC would develop PHLF more easily due to their liver injury of underlying parenchyma (especially caused by cirrhosis and steatosis) and the large extent of liver resection [2]. Therefore, preoperative prediction of PHLF is important for the strategy of treatment. However, there is no gold standard for the prediction.

Indocyanine green (ICG) clearance test is the most commonly used and is considered relatively reliable for liver function reserve assessment [3]. However, this method is not feasible in patients with an iodine allergy or thyrotoxicosis, because ICG contains iodine. Cases of anaphylactic reaction were reported [4]. And the efficacy of ICG clearance test for PHLF prediction was reported to be dissatisfactory [5, 6].

In recent years, two-dimensional shear wave elastography (2D-SWE) for measurement of liver stiffness value (LSV), has been reported high accuracy and reproducibility for quantitative assessment of liver fibrosis [7, 8], and has been adopted for clinical use [9]. On the other hand, in the same stage of liver fibrosis, the presence of severe liver steatosis seems to reveal higher liver stiffness value [10]. On the basis that both liver fibrosis/cirrhosis and severe liver steatosis have great effects on the liver function [2, 11], N Heucke et al. found that liver stiffness measured by 2D-SWE was strongly correlated with parameters of ^13^C-methacetin Liver MAximum capacity (LiMAx) test (a liver function test) [12]. In addition, Shen et al. demonstrated that preoperative liver stiffness measured by 2D-SWE could effectively predict PHLF (AUC = 0.715, *p* < 0.001) [13]. Thus, 2D-SWE may be a promising method for PHLF prediction, with safe, non-invasive, and convenient mode of examination. Another imaging modality for PHLF prediction, Gd-EOB-DTPA-enhanced MRI, demonstrated precise evaluations of regional liver function and estimation of PHLF in smaller study groups [14, 15]. However, this method suffers from limited availability, high cost of time and money.

Furthermore, some serum biomarker panels of diffuse liver diseases and liver function, were also reported for non-invasive and convenient prediction of PHLF. Zou et al. identified albumin-bilirubin (ALBI) scores as an independent predictor of PHLF [16]. In the study of Zhang et al., AUCs of aspartate aminotransferase–platelet ratio index (APRI) and Fibrosis-4 (FIB-4) for preoperative PHLF prediction were 0.697 and 0.696, respectively (*p* < 0.001 for both) [17].

However, the efficacy of different methods for preoperative PHLF prediction has not been compared systematically.

In this study, we aimed to compare the efficacy of preoperative 2D-SWE, ICG clearance test and biomarkers for PHLF prediction in patients with HCC.

## Materials and methods

### Patients

Between February 2019 and December 2019, 334 patients with HCC were retrospectively and consecutively recruited from the general surgery department of our hospital (the diagnosis of HCC was pathologically confirmed after liver resection). All of these patients had adequate data of preoperative assessment (including 2D-SWE examination, ICG clearance test and laboratory testing), operative factors (including hilar occlusion, operative time and blood loss) and post hepatectomy outcomes (including histological inflammation grade and fibrosis stage of underlying hepatic parenchyma, tumor size, parameters of laboratory testing).

The exclusion criteria were: (a) patients who underwent preoperative clinical intervention (for example, transhepatic arterial chemotherapy and embolization/TACE, hepatectomy and chemotherapy) (n = 25), (b) patients with elevated level of total bilirubin (TB, normal range: 5.0–28.0 μmol/L) and/or evident elevated level (five times more than normal limit) of aspartate transaminase (AST, normal range: < 35 IU/L), or alanine transaminase (ALT, normal range: < 40 IU/L) (n = 11), (c) the volume of removed liver were less than 3 segements (n = 73), (d) patients who failed to estimate LSV by 2D-SWE (n = 1), (e) time interval between ICG clearance test, 2D-SWE examination and laboratory testing was more than 2 months (n = 9).

**Fig. 1:**
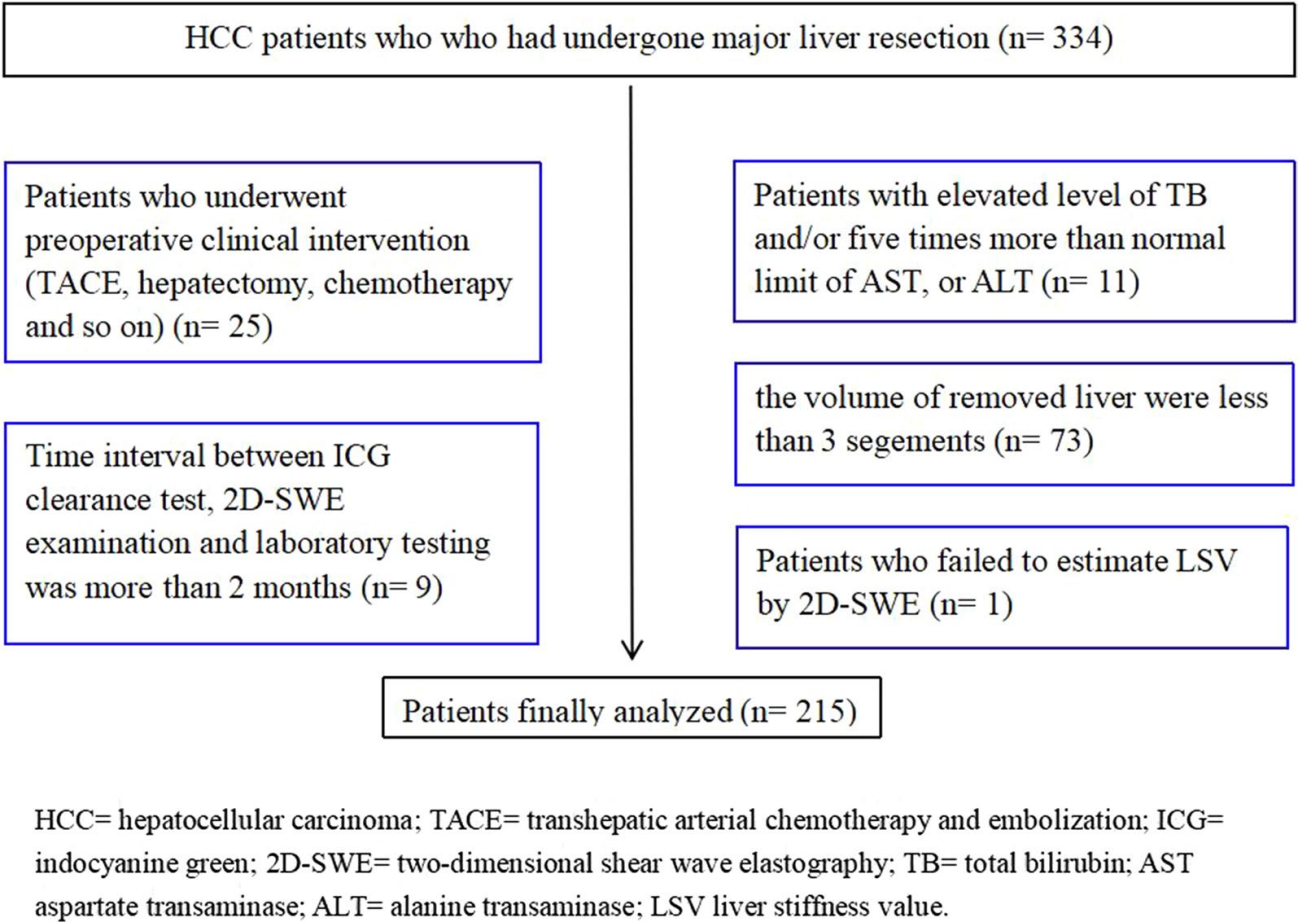
Flow chart of patient selection

Thus, 215 patients were finally analyzed in our study (Fig. 1) (mean age: 54 years, range: 23–78 years). Study patients were divided into two cohorts randomly. In the development cohort (n = 150), the cutoff values of different methods for predicting PHLF were calculated. In the validation cohort (n = 65), validation of the developed cutoff values was performed.

### LSV measurement by 2D-SWE examination

Aixplorer US imaging system (Supersonic Imagine, Axi-en-Provence, France) equipped with a 1-6 MHz transducer (SC6-1), was employed in our study.

LSV measurement was conducted after B-mode and Doppler-mode US examination of the whole liver.

**Fig. 2:**
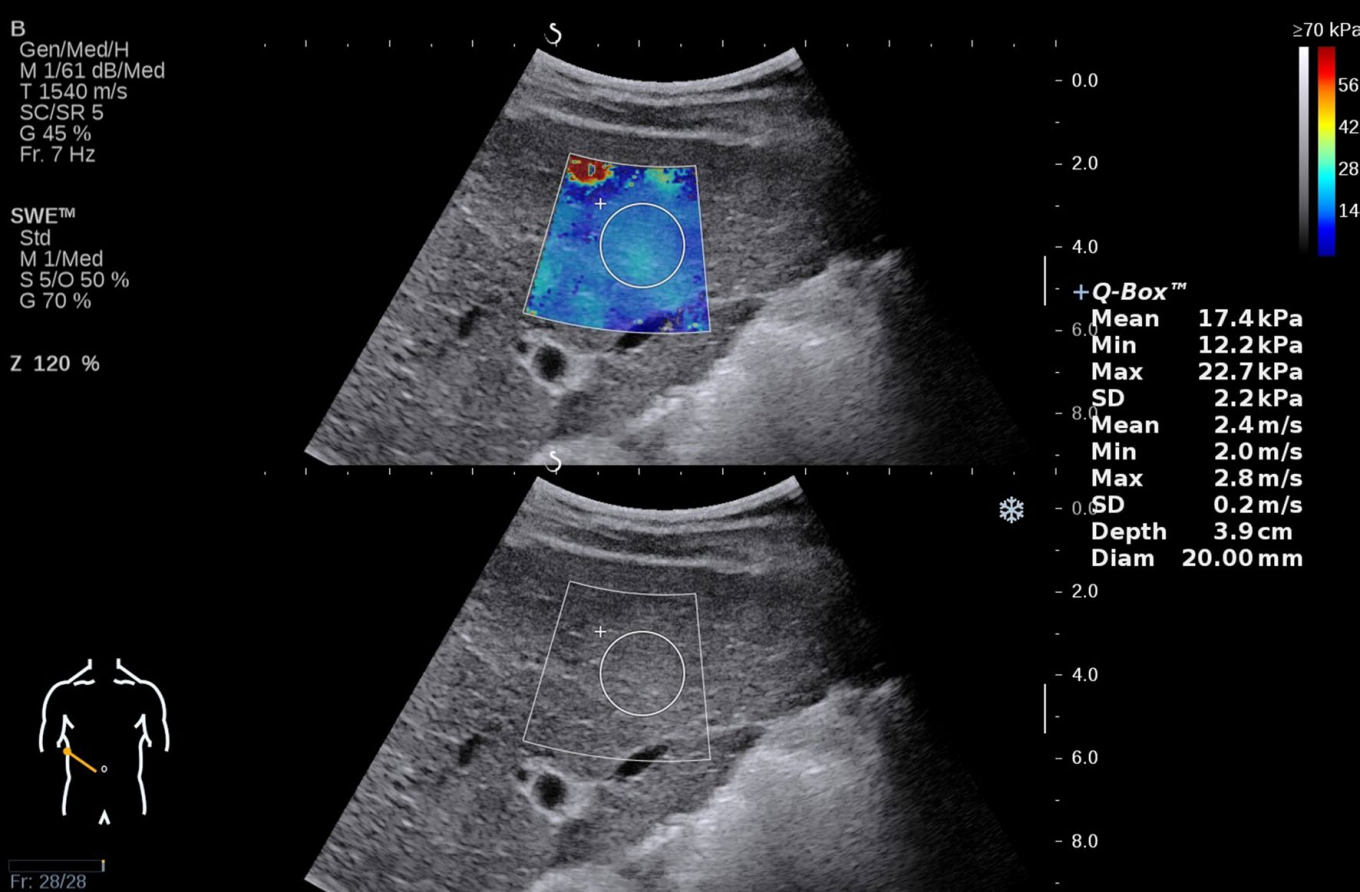
Liver stiffness value (LSV) measured by two-dimensional shear wave elastography (2D-SWE)

Performance of 2D-SWE examination was on the basis of “European Federation for Ultrasound in Medicine and Biology (EFSUMB) Guidelines and Recommendations on the Clinical Use of Ultrasound Elastography” [9]. Patients fasted for more than 6 h before the measurement. They were in supine position, with right arm above the head. The transducer was aligned along a right intercostal space, to illustrate the right lobe of liver in gray scale imaging. An optimal cross-sectional image was considered when no large vessels and no liver masses were observed. Then the patient was asked to hold breath for 3–5 s in natural breathing cycle, and SWE mode was switched on subsequently. A sector region of elasticity imaging (approximately 4 × 3 cm) was located 1–2 cm below the liver capsule. When elasticity signal was stabilized and filled more than 90% of the sector region, a circular region of interest (ROI) with a diameter of 2 cm was placed in the position of homogeneous elasticity signal, and the mean elasticity value (kPa) of ROI was calculated as a result of the measurement (Fig. 2). Failure of measurement was considered if elasticity signal was filled less than 50% of the elasticity imaging region, or minimum value of elasticity in ROI was no more than 1.0 kPa. Five valid results for each patient were acquired, and the median elasticity value of these results was recorded as the data of our study. Patients who were not able to hold their breath for measurement were excluded in our study.

### ICG clearance test

Patients were measured in supine position, with a probe fixed to the nose. A standard dose of ICG (0.5 mg/kg) was prepared for ICG solution of 5 mg/ml (sterile water/ ICG = 5 mL:25 mg). The solution was injected rapidly via median cubital vein, and then ICG-R15 was recorded by DDG-3300 K system (pulse-dye densitometry).

### Serum biomarker panels

Serum biomarkers including AST (IU/L), ALT (IU/L), platelet count (10^9^ /L), age (years), TB (μ mol/L) and albumin (g/L) were used for panels calculation.

$$\begin{array}{*{20}l} {{\text{ALBI score}} = 0.66\times\log_{10} \left( {{\text{TB}} - 0.085\times{\text{albumin}}} \right). } \hfill \\ {{\text{APRI}} = \left( {{\text{AST}}/{\text{upper}}\,{\text{limit}}\,{\text{of}}\,{\text{normal}}\,{\text{AST}}} \right)/{\text{platelet}}\,{\text{count}}\,\times100;} \hfill \\ {{\text{FIB - 4}} = \left( {{\text{age}}\times{\text{AST}}} \right)/\left( {{\text{platelet}}\,{\text{count}}\times\sqrt{AST} } \right).} \hfill \\ \end{array}$$

### Diagnostic criteria

According to the International Study Group of Liver Surgery (ISGLS) criteria, patients with elevated serum values of international normalized ratio (INR) and TB on or after postoperative day 5 are considered a diagnosis of PHLF. Grade A PHLF is diagnosed based on deterioration from the preoperative baseline, which does not require a change in the clinical management. Grade B PHLF results in a deviation from the regular clinical pathway but manageable with noninvasive treatment. Patients who develop PHLF requiring an invasive procedure are classified as having grade C PHLF [18]. It should be specially explained that patients with grade B and C PHLF were divided into “patients with PHLF group” in our study.

Histological inflammation and fibrosis of liver parenchyma were evaluated based on the Scheuer scoring system (published in 1994) [19].

### Statistical analysis

Statistical analysis was performed by SPSS 24.0 and medcalc. Mann–Whitney U test and chi-square test were used to evaluate the significant difference between categories. The area under the receiver operating characteristic (ROC) curve (AUC) was performed for analyzing diagnostic performance, with a calculation of optimal cut-off values (the point that maximized the sum of sensitivity and specificity). Delong test was conducted for comparison between AUCs. Accuracy, sensitivity, specificity were calculated in the validation cohort. Cochran’s Q test was used to compare sensitivity and specificity among groups. Significant difference was considered if *p* < 0.05.

## Results

### Characteristics of study patients

In our study, 215 patients with HCC were finally enrolled. The mean age of study patients was 54 years (range, 23–78 years), and 179 (83.2%) of them were male.

17 patients in the development cohort and 6 patients in the validation cohort developed PHLF.

Basic characteristics of study patients were described including age, gender, body mass index (BMI), platelet count, TB, ALT, AST, albumin, and international normalized ratio of prothrombin time (INR) (Table 1).

No significant difference of operative factors (hilar occlusion, operative time and blood loss), histological inflammation grade and fibrosis stage of underlying hepatic parenchyma, tumor size was found between patients with and without PHLF, neither in the development cohort nor in the validation cohort (Table 1).

### Development cohort

#### LSV, ICG-R15, ALBI scores, APRI, and FIB-4 of patients

The median LSV of patients with PHLF was 13.0 kPa, which was significantly higher than patients without PHLF (8.5 kPa) (*p* < 0.0001). The median ICG-R15 values of patients with PHLF (6.4%) was significantly higher than patients without PHLF (5.2%) (*p* = 0.009). The median ALBI scores of patients with PHLF was -2.5, which was significantly higher than patients without PHLF (-2.8) (*p* = 0.012). And no significant difference of APRI and FIB-4 scores was shown between patients with and without PHLF (Table 2).

#### Performance of preoperative LSV, ICG-R15, ALBI scores for prediction of PHLF

**Fig. 3:**
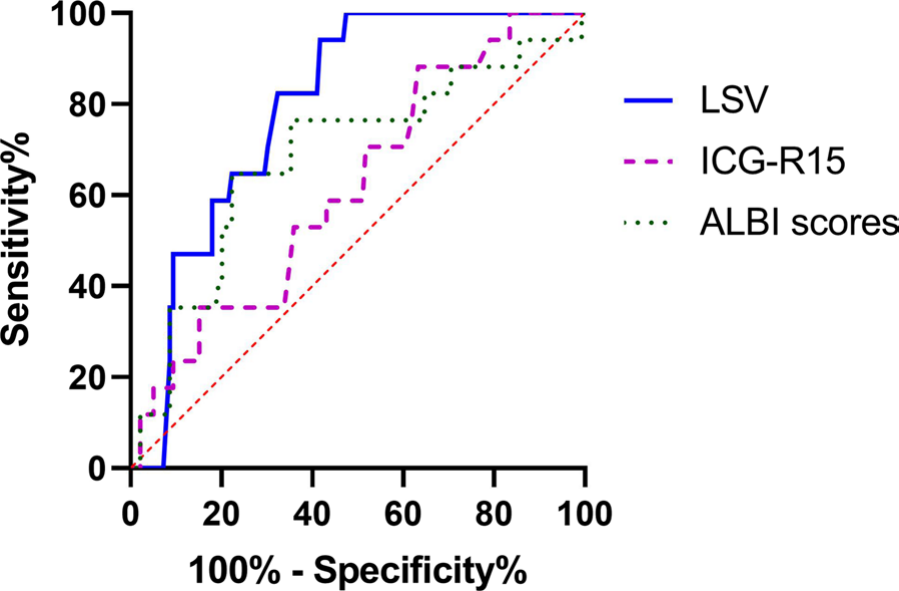
The receiver operating characteristic curves (ROC) of liver stiffness value (LSV), indocyanine green (ICG) retention rate at 15 min (ICG-R15) and albumin-bilirubin (ALBI) scores for predicting post hepatectomy liver failure (PHLF)

The AUC of LSV for predicting PHLF was 0.795, which was significantly higher (*p* < 0.0001) than AUCs of ICG-R15 (AUC = 0.619) and ALBI scores (AUC = 0.686) (Table 3, and Fig. 3).

### Validation cohort

#### Performances of preoperative LSV, ICG-R15, ALBI scores for PHLF prediction

According to analysis of the development cohort, cutoff values of LSV, ICG-R15 and ALBI scores for predicting PHLF were 10.35 kPa, 6.4%, and − 2.547, respectively. Based on the cutoff values of them, LSV revealed higher specificity (76.3%), than that of ICG-R15 (specificity, 66.1%) and ALBI scores (specificity, 69.5%) in the validation cohort (*p* < 0.0001), while showed no significant difference of sensitivity among them (Table 3).

## Discussion

The preoperative prediction of PHLF is essential for treatment and prognosis. However, the efficacy of various preoperative methods for PHLF prediction including ICG clearance test, biomarkers, imaging techniques has not been compared systematically through literature review. This study compared and validated the performance of LSV, ICG-R15, ALBI scores, APRI and FIB-4 for PHLF prediction. In our study, Child-Turcotte-Pugh (CTP) score, a widely used tool for liver function assessment, was not employed. Because CTP score is a classification system, and two of its criteria (ascites and hepatic encephalopathy) are subjective. Previous studies demonstrated the variability and uncertainty of post hepatectomy outcomes among patients with CTP grade A [20]. And noticeably, the patients of our study were all classified as CTP grade A.

In our development cohort, LSV, ICGR15, and ALBI scores revealed significant difference between patients with/without PHLF, whereas no significant difference was found in APRI and FIB-4 scores. Zhang et al. showed that APRI and FIB-4 scores were weak risk factors for PHLF prediction, which was consistent with our results [17]. Therefore, APRI and FIB-4 scores were not included in our further analysis. On the other hand, LSV (AUC = 0.795, *p* < 0.0001) showed higher efficacy for predicting PHLF than ICG-R15 (AUC = 0.619, *p* = 0.109) and ALBI scores (AUC = 0.686, *p* = 0.012). A possible reason for the result is that LSV is an intrinsic property of liver. ICG clearance test is influenced substantially by the hepatic blood flow. Local (including portal hypertension or arterial thrombosis) and general factors (such as circadian variations or low cardiac output) could affect hepatic blood flow and thus ICG clearance [21]. Chong et al. reported higher efficacy of preoperative LSV (AUC = 0.647) for predicting high-grade PHLF than ICG-R15 (AUC = 0.552), which is in keeping with our study [22]. Therefore, LSV could be an alternative method for preoperative prediction of PHLF.

In our validation cohort, the cutoff value of LSV (10.35 kPa) obtained from the development cohort demonstrated significant higher specificity for predicting PHLF than that of ICG-R15 and ALBI scores. Lei et al. indicated that LSV revealed good efficacy for predicting PHLF (AUC = 0.860, *p* < 0.001) and its cutoff value (14.0 kPa) had high sensitivity (94.6%) [23]. They presented a higher cutoff value than our study mainly due to the different diagnostic criteria of PHLF and different measurement equipment of LSV.

There were some limitations in our study. It was a retrospective single center analysis. And the patient numbers of PHLF groups were relatively small due to the low morbidity, which results in the noneffective comparison of sensitivity between LSV, ICG-R15 and ALBI scores and the weaker power of the study. There is a call for a long-term, multicenter and large sample size study to draw further conclusions.

In conclusion, compared with ICG-R15, ALBI scores, APRI and FIB-4, LSV measured by 2D-SWE may demonstrate better capability for predicting PHLF before major liver resection in patients with HCC. Although higher LSV may not have an influence on the operation strategy based on the results of present study, it would be a sign of additional attention and management during perioperative period.

**Table 1:** Characteristics of study patients

Variable	All patients (n = 215)	Development cohort			Validation cohort			P value*
		Patients without PHLF (n = 133)	Patients with PHLF (n = 17)	P value	Patients without PHLF (n = 59)	Patients with PHLF (n = 6)	P value	
Age (years)	54 ± 11	55 ± 10	52 ± 11	0.528	52 ± 9	54 ± 11	0.366	0.556
Numbers of male patients (%)	179 (83.2)	119 (89.5)	15 (88.2)	0.876	51 (86.4)	5 (83.3)	0.834	0.504
Body mass index (kg/m^2^)	23.0 ± 3.2	23.2 ± 3.3	21.5 ± 2.3	0.060	22.7 ± 3.0	22.3 ± 2.7	0.320	0.679
Serum biomarker levels								
Platelet count (10^9^/L)	137	143	147	0.921	138	141	0.098	0.053
TB (μ mol/L)	14.3	14.3	15.1	0.785	14.2	14.7	0.112	0.685
AST (U/L)	34	34	41	0.232	33	40	0.304	0.656
ALT (U/L)	33	33	34	0.787	32	45	0.145	0.966
Albumin (g/L)	42.2	42.2	38.5	0.170	42.7	39.2	0.169	0.521
INR	1.03	1.03	1.05	0.171	1.06	1.04	0.765	0.072
Histological inflammation grade								
G0	20	14	3	0.547	3	0	0.910	0.415
G1	55	35	2		16	2		
G2	87	51	7		26	3		
G3	53	33	5		14	1		
Fibrosis stage								
S0	21	13	1	0.567	7	0	0.670	0.757
S1	10	6	0		3	1		
S2	32	21	1		9	1		
S3	52	30	4		17	1		
S4	100	63	11		23	3		
Tumor size > 5 cm (%)	91 (42.3)	47 (35.3)	9 (52.9)	0.158	31 (52.5)	4 (66.7)	0.508	0.024
Operative factors								
Hilar occlusion (min)	14	14	15	0.378	13	14	0.102	0.067
Operative time (min)	256	256	254	0.138	254	251	0.645	0.122
Blood loss (mL)	300	300	400	0.059	200	300	0.496	0.061

Data were described as means ± standard deviations or medians as appropriate. Comparison between patients with and without post hepatectomy liver failure (PHLF) groups was conducted respectively in the development cohort and validation cohort. Asterisk (*) revealed comparison between development cohort and validation cohort

TB = total bilirubin; AST = aspartate transaminase; ALT = alanine aminotransferase; INR = international normalized ratio of prothrombin time

**Table 2:** Parameters of development cohort

Variable	All patients (n = 150)	Patients without PHLF (n = 133)	Patients with PHLF (n = 17)	P value
LSV (kPa)	9.0 (6.9, 11.9)	8.5 (6.8, 11.0)	13.0 (10.5, 16.0)	< 0.0001
ICG-R15 (%)	5.4 (3.4, 7.2)	5.2 (3.4, 7.1)	6.4 (4.0, 8.8)	0.009
ALBI scores	− 2.8 (− 3.0, − 2.5)	− 2.8 (− 3.0, − 2.6)	− 2.5 (− 2.8, − 2.2)	0.012
APRI	0.63 (0.41, 1.16)	0.62 (0.38, 1.14)	0.62 (0.50, 1.29)	0.488
FIB-4	9.17 (6.81, 11.55)	9.17 (6.79, 11.60)	8.88 (6.76, 11.30)	0.704

Data were described as medians (interquartile range). Statistical analysis was conducted for comparison between patients with and without post hepatectomy liver failure (PHLF) groups. LSV = liver stiffness value; ICG-R15 = indocyanine green retention rate at 15 min; ALBI = albumin-bilirubin; APRI = aspartate aminotransferase–platelet ratio index; FIB-4 = Fibrosis-4

**Table 3:** Liver stiffness, ICG-R15 and ALBI scores in predicting PHLF

Variable	Development cohort					Validation cohort			
	AUC (95% CI)	*P* value	Criterion	Accuracy (%)		Sensitivity (%)	*P* value	Specificity (%)	*P* value
LSV	0.795 (0.714, 0.877)	< 0.0001	> 10.35 kPa	76.9		83.3	0.223	76.3	< 0.0001
ICG-R15	0.619 (0.487, 0.752)	0.109	> 6.4%	66.1		66.7		66.1	
ALBI scores	0.686 (0.541, 0.832)	0.012	> − 2.547	70.8		83.3		69.5	

In the development cohort, AUCs were compared with AUC = 0.5, in the validation cohort, comparison was conducted between LSV, ICG-R15 and ALBI scores. LSV = liver stiffness value; PHLF = post hepatectomy liver failure; ICG-R15 = indocyanine green retention rate at 15 min; ALBI = albumin-bilirubin; NPV = negative predictive value; PPV = positive predictive value

## Data Availability

The datasets analysed during the current study are not publicly available due to conduction of further study but are available from the corresponding author on reasonable request.

## Abbreviations

PHLF: Post hepatectomy liver failure
2D-SWE: Two-dimensional shear wave elastography
ROI: Region of interest
LSV: Liver stiffness value
ICG: Indocyanine green
ICG-R15: Indocyanine green retention rate at 15 min
HCC: Hepatocellular carcinoma
ALBI: Albumin-bilirubin
APRI: Aspartate aminotransferase–platelet ratio index
FIB-4: Fibrosis-4
AUC: The area under the receiver operating characteristic curve
PPV: Positive predictive value
NPV: Negative predictive value
TB: Total bilirubin
AST: Aspartate transaminase
ALT: Alanine transaminase
INR: International normalized ratio
